# Expert panel consensus recommendations for diagnosis and treatment of secondary osteoporosis in children

**DOI:** 10.1186/s12969-020-0411-9

**Published:** 2020-02-24

**Authors:** Rocío Galindo-Zavala, Rosa Bou-Torrent, Berta Magallares-López, Concepción Mir-Perelló, Natalia Palmou-Fontana, Belén Sevilla-Pérez, Marta Medrano-San Ildefonso, Mª. Isabel González-Fernández, Almudena Román-Pascual, Paula Alcañiz-Rodríguez, Juan Carlos Nieto-Gonzalez, Mireia López-Corbeto, Jenaro Graña-Gil

**Affiliations:** 1 UGC Pediatría. Sección Reumatología Pediátrica, Hospital Regional Universitario de Málaga, Málaga, Spain; 2 Unidad de Reumatología Pediátrica, Hospital Sant Joan de Déu, Barcelona, Spain; 3 Servicio de Reumatología, Hospital de la Santa Creu i Sant Pau, Barcelona, Spain; 4 Unidad de Pediatría, Sección Reumatología Pediátrica, Hospital Universitari Son Espases, Palma de Mallorca, Spain; 5 Unidad de Reumatología, Hospital Universitario Marqués de Valdecilla, Santander, Spain; 6 UGC Pediatría, Sección Reumatología Pediátrica, Hospital Campus de la Salud, Granada, Spain; 7 Unidad de Reumatología, Hospital Miguel Servet, Zaragoza, Spain; 8 Unidad de Pediatría, Sección de Reumatología Pediátrica, Hospital La Fe, Valencia, Spain; 9 Unidad de Reumatología, Hospital General de Villalba, Madrid, Spain; 10 Unidad de Pediatría, Sección de Reumatología Pediátrica, Hospital Virgen de la Arriaxaca, Murcia, Spain; 11 Servicio de Reumatología, Hospital General Universitario Gregorio Marañón, Madrid, Spain; 12 Servicio de Reumatología Hospital Vall d’Hebron, Barcelona, Spain; 13 Servicio de Reumatología, Complejo Hospitalario Universitario A Coruña, A Coruña, Spain; 14 Osteogenesis Imperfecta and Secondary Osteoporosis Working Group from the Spanish Pediatric Rheumatology Society, Madrid, Spain

**Keywords:** Secondary osteoporosis, Children, Low bone mineral density

## Abstract

**Background:**

Osteoporosis incidence in children is increasing due to the increased survival rate of patients suffering from chronic diseases and the increased use of drugs that can damage bones.

Recent changes made to the definition of childhood osteoporosis, along with the lack of guidelines or national consensuses regarding its diagnosis and treatment, have resulted in a wide variability in the approaches used to treat this disease.

For these reasons, the Osteogenesis Imperfecta and Childhood Osteoporosis Working Group of the Spanish Society of Pediatric Rheumatology has sounded the need for developing guidelines to standardize clinical practice with regard to this pathology.

**Methods:**

An expert panel comprised of 6 pediatricians and 5 rheumatologists carried out a qualitative literature review and provided recommendations based on evidence, when that was available, or on their own experience.

The level of evidence was determined for each section using the Oxford Centre for Evidence-based Medicine (CEBM) system. A Delphi survey was conducted for those recommendations with an evidence level of IV or V. This survey was sent to all members of the SERPE. All recommendations that had a level of agreement higher or equal to 70% were included.

**Results:**

Fifty-one recommendations, categorized into eight sections, were obtained. Twenty-four of them presented an evidence level 4 or 5, and therefore a Delphi survey was conducted. This was submitted electronically and received a response rate of 40%. All recommendations submitted to the Delphi round obtained a level of agreement of 70% or higher and were therefore accepted.

**Conclusion:**

In summary, we present herein guidelines for the prevention, diagnosis and treatment of secondary childhood osteoporosis based on the available evidence and expert clinical experience. We believe it can serve as a useful tool that will contribute to the standardization of clinical practice for this pathology. Prophylactic measures, early diagnosis and a proper therapeutic approach are essential to improving bone health, not only in children and adolescents, but also in the adults they will become in the future.

## Background

Osteoporosis is a disorder characterized by bone mass reduction and alterations in the micro-architecture of the bone tissue resulting in bone fragility and, consequently, an elevated risk of fractures [1]. At present, osteoporosis is a public health problem in industrialized countries affecting about 30% of women and 8% of men older than 50 in Europe [2]. Although it has typically been considered an adult disorder, it is becoming increasingly clear that osteoporosis might be rooted in childhood and adolescence [3]. Bone matrix mineralization takes place during these stages of life, and therefore subjects reach peak bone mass at the end of this growth phase. If this peak is not optimal, it will facilitate the development of osteoporosis in adulthood [3].

Furthermore, the incidence of childhood osteoporosis is increasing due to, among other factors, the increased survival rate of patients suffering from chronic diseases and the increased use of drugs that can damage bones [4].

In its Official Positions published in 2013, the International Society for Clinical Densitometry considered two possible conditions regarding the diagnosis of childhood osteoporosis [5]:

- Presence of one or more vertebral fractures (VF) in the absence of local disease or high-energy trauma.
- Z-score of bone mineral density (BMD) or bone mineral content (BMC) ≤ − 2 (adjusted for size in cases of children measuring below the 3rd percentile) and a history of clinically significant fractures; specifically:
  - two or more long bone fractures occurring by age 10 years; or
  - three or more long bone fractures at any age up to age 19 years.

Moreover, their guidelines state that a Z-score higher than -2 does not preclude the possibility of bone fragility, especially in those patients suffering from disorders that favor secondary osteoporosis.

At present, in pediatrics there is growing interest in this pathology. However, recent changes made to the definition of childhood osteoporosis [5, 6], along with the lack of guidelines or national consensuses regarding its diagnosis and treatment, have resulted in a wide variability in the approaches used to treat this disease [7].

For these reasons, the Working Group of Osteogenesis Imperfecta and Childhood Osteoporosis of the Spanish Society of Pediatric Rheumatology (SERPE, by its Spanish acronym) has sounded the need for developing guidelines to standardize clinical practice with regard to this pathology.

## Methods

### Design

A qualitative synthesis of scientific evidence and consensus based on clinical experience and existing scientific evidence was used to formulate the study design.

### Development stages

Preparation began with the establishment of an expert panel comprised of 11 physicians, 6 pediatricians and 5 rheumatologists. All are members of the SERPE and have experience in the diagnosis and treatment of secondary osteoporosis in children.

Study coordination was undertaken by one of the physicians.

This panel of experts reached a consensus on the essential contents to include in the document, consisting of the following eight sections:

1. When osteoporosis should be suspected
2. How to prevent it
3. Laboratory tests used in screening
4. Imaging tests used in screening
5. Treatment: calcium and vitamin D supplementation
6. Treatment: bisphosphonates (BPs)
7. Follow-up
8. Glucocorticoid-induced osteoporosis (GIOP)

One or two experts were appointed to be responsible for a literature review of each section, which was conducted with the assistance of a rheumatologist and an expert in methodology.

Following the revision, each of the experts responsible for the literature review provided recommendations regarding each section based on evidence, when that was available, or on their own experience.

Afterwards, the panelists held a meeting to discuss and draft the recommendations. The level of evidence was determined for each section using the Oxford Centre for Evidence-based Medicine (CEBM) system. For this purpose, both a rheumatologist and an expert in methodology provided guidance. A Delphi survey was conducted for those recommendations with an evidence level of IV or V. This survey was sent to all members of the SERPE. All recommendations that had a level of agreement higher or equal to 70% were included.

## Results

A total of 51 recommendations, categorized into eight sections, were obtained (Table 1). Twenty-four of them presented an evidence level 4 or 5, and therefore a Delphi survey was conducted. This was submitted electronically and received a response rate of 40%. All recommendations submitted to the Delphi round obtained a level of agreement of 70% or higher and were therefore accepted.

**Table 1:** Recommendations, levels of evidence, grade of recommendation according to the Oxford CEBM and level of agreement in Delphi round*

	Recommendation	LE	GR	LADR
General recommendations				
1	There is a need to monitor BMD in patients with chronic diseases, especially those of endocrinologic, nutritional, rheumatological, renal, metabolic, hematological, neurological and gastrointestinal origin. There is no universal consensus regarding when and how to carry out such an assessment for all the pathologies involved. Following the existing guidelines for each pathology is therefore recommended [8–22].	4	D	95%
2	Special attention must be paid to patients with chronic diseases who receive treatments that contribute to osteoporosis development; e.g., GCs, chemotherapy treatments or antiepileptic drugs [17, 19, 20, 23–28].	2b	B	
Lifestyle and dietary habits				
3	It is important to identify those children at risk for osteoporosis due to causes related to lifestyle, long-term immobilization or problems of anorexia or malnutrition [19, 29–34].	2a	B	
4	Healthy dietary habits must be established by means of a balanced diet that meets specific calcium and vitamin D requirements for each age and allowing for adequate nutritional intake [35, 36].	2a	B	
5	Tobacco, caffeine and alcohol use must be avoided in children and adolescents [35, 37, 38] [39–41].	2a	B	
6	High impact and low frequency exercise - e.g., running or jumping - must be recommended for healthy children and adolescents [31, 42–46].	1a	A	
7	This kind of sport must also be recommended for children with low BMD [23, 47, 48].	3a	C	
8	It is important to recommend exposure to sunlight on the hands, face and arms between six to eight minutes/day in the summer (avoiding the hottest part of the day between 10 a.m. and 3 p.m.) and 30 min/day during the coldest months of the year [15, 49, 50].	4	D	90%
Complementary test				
9	Children with chronic diseases are at greater risk of vitamin D deficiency than the general population. Therefore, it would be advisable to monitor them, mostly during the end of winter [49–51].	2a	B	
10	Regarding all children and adolescents who are suspected of suffering from secondary osteoporosis, the following lab work for the initial study is recommended: blood and urine chemistry, urine screening and bone turnover markers (Table 6) [52].	5	D	83%
11	The role of bone turnover makers in pediatric populations with bone fragility is insufficiently defined, but they can be useful in treatment follow-up and evaluation. For this reason, they are included in the initial study of these patients [23, 52–56].	5	D	80%
12	Measurements of bone turnover makers in urine are not recommended for all patients since the collection of the sample may be not correctly carried out due to the patient’s age or concomitant disease [57].	5	D	90%
13	Specific complementary tests detailed in Table 7 can be carried out for some children and adolescents depending on the suspected underlying condition [15, 23, 58].	5	D	90%
14	Interpretation of analytical values must be done taking into consideration the factors that modify them. These factors are biological (age, sex, pubertal stage, ethnicity) and other controllable variables (circadian rhythm, diet, season of the year) [53, 55–61].	2b	B	
15	Since low bone mass has been associated with increased fracture risk, DXA is the most recommended method for pediatric populations in order to assess bone health. However, it does not allow for a prediction of fracture risk [34, 48, 62, 63].	2a	B	
16	DXA of the lumbar spine and total body less head (TBLH) is the chosen method to measure BMD in pediatric populations, since they are the most accurate and reproducible areas of the skeletal system for this measurement [6, 64, 65].	2a	B	
17	Data analyses must be carried out using pediatric software (software for adults overestimates BMD) [66, 67].	2b	B	
18	Vertebrae DXA measurement is recommended for children under the age of five due to its higher reproducibility and shorter time necessary for conducting this test [5, 6, 64, 66, 67].	2a	B	
19	For children under the age of three, total body BMD is not recommended on a routine basis due to its lack of reproducibility in such young children; rather BMC should be used [5, 6, 64].	3a	C	
20	Regarding children of short stature (< p3) or with a growth retardation problem, it is recommended to adjust their results by means of a size Z-score [6, 23, 64, 68, 69].	2a	B	
21	For children suffering from joint contracture or with mobility problems - e.g., with cerebral palsy - distal femur measurement can be an alternative [68].	2a	B	
22	For children with suspected secondary osteoporosis, it is recommended to extend the study with a plain lateral thoracic and lumbar X-ray to assess vertebral compression fractures, particularly if they are receiving GCs [23, 70–73].	2b	B	
23	In the event of low bone mass or risk factor persistence, a second plain lateral thoracic and lumbar X-ray should be taken after one year [70, 71] .	3b	C	
24	The minimum time interval to wait before repeating a bone density measurement is six months, a period of one year being advisable, apart from exceptional cases [6, 64, 66, 74, 75].	3b	C	
25	DXA can be used to assess treatment response after six months in the event of high doses of corticosteroids, chemotherapy, or in situations of malnutrition or active treatment [6, 64, 66, 74].	3b	C	
26	In cases of children who initially present normal densitometry results, but in whom risk factor(s) persist, the periodicity of the densitometry must be individualized according to the risk factor associated and an interval of one or two years is advised until peak bone mass is reached [6, 64, 66, 74, 75].	5	D	95%
Prevention				
27	Oral calcium supplementation could improve BMD in healthy children with a low-calcium diet. Nevertheless, increasing calcium intake by means of calcium-rich foods is preferable to supplementation [36–38, 76].	5	D	90%
28	With respect to children with chronic diseases, adequate treatment of the disease is the most important step to be taken regarding osteoporosis prevention and treatment [23, 77–79].	2b	B	
Treatment				
29	Vitamin D supplementation must be prescribed for all those patients with chronic pathologies presenting levels lower than 20 ng/mL and for those with levels between 20-30 ng/mL who present Z-score ≤ − 2 or any data showing bone fragility [51, 80, 81].	4	D	90%
30	For children and adolescents with a low BMD or osteoporosis, calcium supplementation is recommended, particularly for those patients with a low-calcium diet, as well as supplementation of the proper amount of vitamin D_3_ in order to keep plasmatic levels of 25-hydroxyvitamin D_3_ higher than 30 ng/dL [82, 83].	2b	B-C	
31	The required amount of calcium and vitamin D supply needed in children with pathologies that can jeopardize intestinal absorption or modify their body’s use of these nutrients is unknown. For this reason, in the event that such patients present osteoporosis or low BMD according to chronological age, it is advisable to initially prescribe the dose required to ensure a recommended daily intake of healthy children. Treatment can be modified according to plasmatic 25-hydroxyvitamin D_3,_ iPTH and calciuria levels, which must be monitored every six to twelve months [49–51, 82, 83].	5	D	90%
32	Treatment with BP should be administered to those pediatric patients with osteoporosis (Z-score ≤ − 2 + pathological fracture or VF regardless of Z-score) [9, 84–88].	1b	A	
33	Treatment with BP can be considered for patients without osteoporosis, but a low BMD in early puberty (Tanner 2): - When active risk factors are present: patients with Z ≤ − 2. 5 SD (with a declining trajectory confirmed at least on two separate occasions with one year apart). - When patients no longer present active risk factors: patients with Z ≤ -3DS (with a declining trajectory confirmed on at least on two separate occasions with one year apart) [9, 84–87].	5	D	78%
34	Intravenous BPs should be used whenever there are VF, if there is some contraindication to the use of oral BPs, or according to the patient’s preferences [88–91].	3a	B-C	
35	Oral BPs can be used in the absence of contraindications and VF, or during the de-escalation phase [9, 84–87].	5	D	70%
36	The BP dosage should be discontinued or progressively reduced in those patients not presenting fractures during the preceding year and having reached a Z-score higher than -2 [9, 84–87].	5	D	90%
Follow-up				
37	A follow-up is recommended for patients at risk for osteoporosis while other risk factors persist and during treatment with calcium and/or vitamin D_3_, BPs or other osteoporosis treatments [49, 51, 74].	4	D	95%
38	Calcium and phosphorus metabolism (serum levels of calcium, phosphorus, alkaline phosphatase, iPTH and 25-hydroxyvitamin D_3_) should be evaluated on an annual basis [49, 51].	4	D	90%
39	During treatment with vitamin D, it is recommended to monitor serum levels of 25-hydroxyvitamin D_3_ every 6 to 12 months, unless the dosage is changed. In such cases, patients should be monitored at 3–6 months [49, 51].	5	D	88%
40	During supplementation with calcium and/or vitamin D_3_, calcium/creatinine levels in urine should be monitored at least once a year. Renal ultrasounds should be conducted to rule out nephrocalcinosis in the event of calciuria increase, or when it is not possible to determine calciuria due to the patient’s age or pathology [49–51, 82, 83].	5	D	83%
41	DXA is recommended one year after the baseline DXA, and then subsequently every 1 or 2 years depending on the trajectory observed. The minimum interval should be 6–12 months [74].	4	D	93%
42	It is recommended to perform simple lateral thoracic and lumbar X-rays to assess VF every 6 months to 2 years (with 1 year being the average), according to the risk factor magnitude and the functional status of the child [51, 71, 74].	5	D	73%
43	For pediatric patients with reduced mobility due to cerebral palsy and congenital myopathies, a spine X-ray is recommended at 6–8 years of age, or earlier in the event of back pain, and then periodically until the end of growth [23].	5	D	88%
44	During treatment with intravenous BPs, assessments of laboratory parameters are recommended before each administration. For oral BPs, checks every six months are recommended [9, 84–86, 88–92].	5	D	83%
45	During treatment with BPs, annual DXA is recommended [9, 84–86, 88–92].	5	D	85%
Corticosteroid-induced osteoporosis				
46	Lateral spine x-ray is recommended in order to detect VF at the beginning of treatment with GCs and after one year [70, 71, 93].	2a	B	
47	It is recommended to carry out lumbar spine or TBLH DXA within the first six months after the beginning of treatment with GCs, and then every 9 to 12 months if treatment continues [94].	4	D	85%
48	It is recommended to start simultaneous treatment and/ or optimize calcium intake (500–1000 mg/day) and vitamin D 400 IU/day for those patients who are scheduled to receive systemic GCs for three months or more [95].	2b	B	
49	Treatment with calcium and vitamin D must be maintained for three months after discontinuation of GCs [95].	5	D	88%
50	For children and adolescents receiving GCs chronically and presenting low BMD (Z-score ≤ − 2) and pathological fractures, it is recommended to use BPs associated with calcium and vitamin D [87, 95, 96].	1b	A	
51	Lateral spine x-rays or BMD checks with DXA are not recommended on a routine basis for those children and adolescents being treated with inhaled GCs at dosages under 800 mcg/day, unless they present other risk factors [97–99].	1b	A	

*LE* level of evidence*, GR* grade of recommendation*, LADR* Level of agreement in Delphi round*, GCs* glucocorticoids*, BMD* bone mineral density*, BMC* bone mineral content*, DXA* dual-energy x-ray absorptiometry*, iPTH* intact parathyroid hormone*, BPs* bisphosphonates*, VF* vertebral fractures

## Discussion

### When osteoporosis should be suspected

Factors that contribute to osteoporosis in children and adolescents can be both genetic and lifestyle associated.

In children suffering from chronic diseases or receiving bone harmful treatments for a prolonged period of time, several factors that increase bone resorption and decrease bone formation converge and result in increased bone fragility [100] [63]. For this reason, bone health must be assessed during follow-up, adopting adequate preventive measures.

Table 2 shows some of the pathologies responsible for secondary osteoporosis.

**Table 2:** Causes of secondary osteoporosis

Neuromuscular disorders	Cerebral palsy Duchenne muscular dystrophy Rett syndrome Myopathies Diseases resulting in long-term immobilization
Hematological diseases	Leukemias Hemophilia Thalassemia
Systemic autoimmune diseases	Juvenile systemic lupus erythematosus Juvenile dermatomyositis Systemic juvenile idiopathic arthritis Systemic sclerosis
Lung diseases	Cystic fibrosis
Gastrointestinal diseases	Celiac disease Inflammatory bowel disease Chronic liver disease Cow’s milk protein allergy
Renal diseases	Nephrotic Syndrome Chronic renal failure
Psychiatric illnesses	Anorexia nervosa
Infectious diseases	HIV infection Immunodeficiencies
Endocrine diseases	Delayed puberty Hypogonadism Turner syndrome Klinefelter Syndrome Growth hormone deficiency Acromegaly Hyperthyroidism Diabetes Hyperprolactinemia Cushing syndrome Adrenal insufficiency Hyperparathyroidism Vitamin D metabolism disorders
Inborn errors of metabolism	Glycogen storage disease Galactosemia Gaucher disease
Skin conditions	Epidermolysis bullosa
Iatrogenesis	Systemic glucocorticoids Cyclosporine Methotrexate Heparin Anticonvulsants Radiation therapy

There is no universal consensus regarding when and how to assess bone health for all of the pathologies involved. However, there are some clinical guidelines for different pediatric disorders (Table 3).

**Table 3:** Assessment of BMD for certain diseases or chronic treatments involved in childhood secondary osteoporosis

Disease / Treatment	BMD assessment
Celiac disease	DXA if: -no adequate dietary adherence -irregular menstruation -anemia -other risk factors for fractures [74]
Cerebral palsy	Difficult lumbar spine X-ray interpretation in cases of severe scoliosis. Total-body or distal femur DXA (area with higher fracture risk), only if there are fragility fractures [8].
Duchenne muscular dystrophy	Baseline DXA and annual monitoring. Lateral spine x-ray: Baseline - On GCs treatment: Repeat every 1–2 years. - Not on GCs treatment: Repeat every 2–3 years. - If back pain or ≥ 0, 5 SD decline in spine BMD Z score on serial measurements over 12-month period: Repeat. Refer to osteoporosis specialist following the first fracture [11].
Rett syndrome	Baseline DXA, and serial controls according to individual risk [15].
Epilepsy	Consider DXA for epileptic patients receiving anti-epileptic drugs for a prolonged period [13]
Thalassemia	DXA every 2 years from adolescence [12]
Inflammatory/ systemic disease	Consider DXA for patients receiving high doses of GCs [74].
Juvenile idiopathic arthritis (JIA)	< 6 years: DXA in the presence of fragility fractures. > 6 years: DXA if not presenting rapid remission of JIA or in need of high doses of GCs [18].
Neoplasms	Baseline DXA two years after completing chemotherapy with osteotoxic drugs; e.g., MTX, GCs or hematopoietic cells transplantation; or secondary effects that favor osteoporosis development (growth hormone deficiency, hypogonadism, etc.) DXA follow-up based on the results of baseline DXA and persistent risk factors [17]
Cystic fibrosis	DXA in children ≥ age 8 if: - weight < 90% ideal weight - FEV_1_ < 50% - Delayed puberty - High dosis of GCs > 90 days per year At 18, all of them [101].
Diabetes mellitus	DXA if: - low BMD specific risk factors - increased daily insulin dosis - impaired renal function - fracture history [74]
Anorexia nervosa	DXA in patients with amenorrhea for more than 6 months [13].
Systemic lupus erythematosus	DXA evaluation in patients with prolonged systemic GCs exposure exceeding ≥0.15 mg/kg daily for ≥ 3 months. Repeat on an annual basis if Z-score ≤ − 2 [102].

*DXA* dual-energy x-ray absorptiometry*, BMD* bone mineral density*, GCs* glucocorticoids*, MTX* methotrexate*, FR* risk factors

BMD in patients with chronic diseases should be monitored based on the existing guidelines for each disorder. In addition, special attention must be paid to patients suffering from chronic diseases and receiving treatment that may favor the development of osteoporosis; e.g., glucocorticoids (GCs), chemotherapy or antiepileptic drugs.

### How to prevent osteoporosis

There are numerous factors that impact bone health in children. Many of these are modifiable, at least in part (Table 4) [35].

**Table 4:** Risk factors of osteoporosis in childhood

Modifiable	Nutritional	• Caloric intake • Protein intake • Calcium intake • Phosphorus intake • Vitamin D • Others (vitamins K, group B, Mg, K …)
	Lifestyle	• Solar exposure • Physical exercise • Tobacco • Alcohol
Partly modifiable	High risk diseases	• Prematurity • Pregnancy and nursing in adolescents • Intestinal malabsorption • Cystic fibrosis • Celiac disease • Inflammatory bowel disease • Food allergies • Chronic lactose intolerance • Chronic liver disease • Chronic kidney disease • Cerebral palsy • Chronic rheumatic diseases …
	Hormonal	• Treatment with glucocorticoids • Hyperparathyroidism • Hypogonadism
Non- modifiable	Genetics	
	Sex	
	Ethnicity	

Nutritional factors with higher evidence of conferring a positive effect on bone health are calcium, phosphorus and vitamin D [23]. Table 5 shows the daily nutrient requirements for a healthy child [103], although children suffering from chronic diseases or under treatment with drugs that alter intestinal absorption may need higher intakes of calcium and vitamin D [23, 35, 49, 104]*.*

**Table 5:** Daily calcium and vitamin D requirements according to age

Age	Calcium (mg)	Vitamin D (IU)
0–6 months	200	400
6–12 months	260	400
1–3 years	700	600
4–8 years	1000	600
9-18 years	1300	600

Calcium-rich foods are preferable to supplements for attaining optimal calcium intake, not only because they have a higher bioavailability [23] and are easier to digest, but also because their consumption avoids possible secondary cardiovascular effects common in adults [105] and favors positive nutritional habits from childhood [23]. Thus, systematic supplementation with calcium in the absence of osteoporosis or low BMD is not recommended [106]. It should only be considered for those patients with calcium-poor diets [42].

Vitamin D is an important hormone for the absorption and use of calcium [103]. There is some dispute regarding the optimal levels of vitamin D, although in general terms, serum levels of 25-hydroxyvitamin D_3_ ≥ 50 nmol/L (20 ng/mL) are considered normal. Those between 30-50 nmol/L (12-20 ng/mL) are regarded as insufficient, and those < 30 nmol/L (12 ng/mL) deficient [49].

In order to maintain appropriate levels, the intake of food enriched in vitamin D is important, as well as daily exposure to sunlight on the hands, face and arms for 6 to 8 min in the summer months (avoiding the hottest part of the day) and 30 min in the coldest months of the year. However, there is no exposure to ultraviolet B (UVB) rays that is safe in terms of skin cancer [49]*.*

Children suffering from chronic diseases have an increased risk of vitamin D deficiency. Therefore, monitoring vitamin D serum levels would be advisable in these patients, especially in late winter [104].

In addition, a large number of other nutrients play a significant role in bone metabolism, such as proteins, potassium, magnesium, copper, iron, phosphate, zinc and vitamin A, C and K [23, 104]. Thus, it is important to recommend a varied diet, including fruits and vegetables, to ensure an adequate intake of key nutrients in order to maintain good bone health in children and adolescents [23, 35, 104].

Moreover, exercise and regular physical activity are considered among the most effective strategies for maximizing peak bone mass during childhood [23, 35, 42, 104]. High impact and low frequency exercise - e.g., jumping, running or resistance training - favor BMD increase in children and are more suitable than others such as swimming or biking in terms of bone health [35, 43, 104]. Nevertheless, physical activity with excessive impact increases fracture risk [44].

There are other factors such as tobacco, caffeine and alcohol consumption that are associated with decreased BMD and increased fracture risk [35, 37, 38, 104]. This is one reason, among others, to avoid their consumption in children and adolescents.

It is also essential to maintain an adequate nutritional state because both, extreme thinness and adiposity, are associated with lower BMD and increased fracture risk [29, 30, 35].

Furthermore, the optimal control of the primary disease is the most effective way to prevent and treat secondary osteoporosis [23, 35].

### Diagnosis

#### Laboratory tests

The diagnosis of secondary osteoporosis is usually made after the diagnosis of the underlying disease that causes it. However, in some cases, it may be the first manifestation of the underlying disease. Although most of the disorders included in the differential diagnosis can be inferred by means of a thorough medical history review and physical examination, some pathologies - e.g., phosphocalcic metabolism alterations, hypothyroidism or some types of leukemia- can be paucisymptomatic and require complementary tests for accurate diagnosis [100]. For this reason, it is recommended to perform the analytical parameters listed in Table 6 when assessing a child with suspected or established diagnosis of secondary osteoporosis. Regarding the parameters shown in Table 7, they are only justified in the event of clinical suspicion.

**Table 6:** Basic Diagnostic Studies

Laboratory test	Variables to analyze
Blood count	
Blood chemistry	Calcium, ionized calcium, phosphorus, magnesium, total proteins, creatinine, urea, glucose, 25-hydroxyvitamin D_3_, PTH, TSH, free T4
24-hour urine chemistry	Calcium, phosphorus, creatinine, tubular phosphorus reabsorption, sodium
Urine screening	Ca/Creatinine^a^
Bone turnover makers	Total alkaline phosphatase

^a^Sample from a single urination, preferably first one in the morning

**Table 7:** Analytical determinations to make based on suspicion

	Studies
1	Immunoglobulins
2	Anti-transglutaminase IgA antibodies
3	Cortisol
4	Prolactin
5	FSH, LH, testosterone
6	Homocysteine
7	Genetic studies (genes related to osteogenesis imperfecta and disorders characterized by bone fragility)

These biochemical parameters must be interpreted based on factors such as age, sex, growth rate, nutritional status and pubertal stage, among others [52, 59].

Bone turnover markers are certain substances released into the bloodstream during bone formation or resorption that reflect bone metabolic activity at a given time. Though numerous, amino-terminal propeptides from type 1 procollagen (P1NP) and carboxy-terminal telopeptides (CTx) should be used as reference markers to evaluate formation and resorption, respectively [107, 108]. These can be measured in the blood and urine [109], although for children it is preferable to determine them in plasma [53, 110, 111]. In adults, they have been shown to be useful for monitoring treatment in patients with osteoporosis [112]. In children, however, such interpretation is much more complex [54, 110, 113], although they can help in monitoring antiresorptive therapy compliance and measuring its effectiveness [100].

#### Imaging tests

The diagnosis of childhood osteoporosis is essentially based on the presence of fragility fractures. However, a dual-energy x-ray absorptiometry (DXA) is recommended to ensure a complete assessment of bone health [5].

Despite numerous limitations [100], DXA is the chosen method for determining bone health in children. Lumbar spine and total-body less head are the preferred skeletal sites for performing DXA as they are the most accurate and reproducible areas in children. In addition, Z-score should be adjusted according to the height in children with a size below the 3rd percentile [5].

Other techniques for assessing bone quality in pediatrics are peripheral quantitative computed tomography and ultrasound. However, although superior to DXA in certain aspects, there are insufficient studies of pediatric populations to recommend their use on a routine basis [100].

Furthermore, in a patient with suspected or confirmed bone fragility, the presence of VF, which are frequently asymptomatic, should always be assessed by means of a simple lateral full spine x-ray or by DXA vertebral fracture assessment, if feasible [39, 114].

### Treatment

#### Calcium and vitamin D supplementation

Calcium and vitamin D supplementation have not shown any clinically significant effect on BMD in studies performed in healthy children [106]. In contrast, some studies have reported a favorable effect in patients with chronic diseases that favor osteoporosis such as cerebral palsy [8]. On the other hand, no side effects have been reported [8, 40]. Thus, although there are no studies that assess the effect of supplementation on the incidence of fractures, calcium supplementation is considered advisable in children and adolescents with low BMD or osteoporosis, especially those patients with a low dietary intake.

Likewise, ensuring proper vitamin D_3_ intake is recommended in order to maintain plasmatic levels of 25-hydroxyvitamin D_3_ higher than 50 nmol/l (20 ng/dL).

Table 6 shows the recommended daily intake of calcium and vitamin D for healthy children [103]. The optimal intake for children with disorders that may interfere with intestinal absorption or modify calcium metabolism remains unknown [41]. Thus, initially, supplementation should be prescribed with respect to these recommendations and subsequently be modified according to plasmatic 25-hydroxyvitamin D_3,_ intact paratohormone (iPTH) and calciuria, which must be monitored every 6–12 months.

#### Bisphosphonates

BPs are synthetic analogs of pyrophosphate that inhibit bone resorption. They selectively concentrate and increase BMD in high remodeling rates skeleton areas [115]. They are hydrophilic drugs with low intestinal absorption (< 1%) and high distribution volumes that are excreted in urine. Thus, dosages must be adjusted according to glomerular filtrate. Moreover, they are characterized by a very slow elimination from bone tissue, and remain in the body for years after treatment [115].

Knowledge on the mid- and long-term safety of these drugs is constantly increasing [100]. Thus, some authors recommend their use as long as osteoporotic criteria are met, particularly in those patients with long bones and VF and who exhibit poor potential for spontaneous recovery (age at puberty, risk factor persistence, etc.) [116].

To date, BPs have only been prescribed as a secondary prevention measure. In other words, once the first fracture occurs. Their use is intended to prevent the appearance of new fragility fractures. It is currently known that they confer a positive effect on BMD [9, 84–87], and there is increasing knowledge regarding their long-term safety [100]. On the other hand, if the peak bone mass reached at the end of the growth stage is not optimal, osteoporosis is more likely to develop during the later stages of life [3]. On the basis of the above data, our working group recommends that clinicians consider treatment with BPs for those patients without osteoporosis, but low BMD in early puberty, with low Z-scores and decreasing trajectories.

In any case, BPs are used off label in childhood osteoporosis, so informed consent must be obtained when they are prescribed.

Second and third generation BPs are the most commonly used BPs in children. Some of them are intravenously administered and others orally [100]. Oral BPs are widely used in adulthood osteoporosis, and some studies have demonstrated that they increase BMD and decrease fracture risk in patients with Osteogenesis Imperfecta. Nevertheless, in contrast to intravenous BPs, they lack sufficient potency to induce remodeling after VF [117] and are contraindicated in patients with esophagitis risk factor; e.g., gastroesophageal reflux or hiatal hernia. Intravenous BPs are preferred for pediatric osteoporosis, and oral BPs are only used for patients with mild forms of osteoporosis, without VF, when intravenous administration is contraindicated for any reason, or during the treatment maintenance phase [100].

Table 8 shows doses and dosing intervals for the most commonly used BPs in pediatrics [100].

**Table 8:** Doses and dosing intervals for the most commonly used BPs in pediatrics

Drug	Administration	Dose
Pamidronate (2nd generation)	Intravenous (dilute in 100-250 ml physiological saline solution, in 3–4 hours)	< 1 year: 0. 5 mg/kg every 2 months 1–2 years: 0. 25-0. 5 mg/kg/day 3 days every 3 months 2–3 years: 0.375–0.75 mg/kg/day 3 days every 3 months > 3 years: 0. 5–1 mg/kg/day 3 days every 4 months Maximum dose: 60 mg/dose and 11. 5 mg/kg/year
Neridronate (3rd generation)	Intravenous (dilute in 200–250 ml physiological saline solution, in 3 hours)	1–2 mg/kg/day every 3–4 months
Zolendronate (3rd generation)	Intravenous (dilute in 50 ml physiological saline solution, in 30-45 min)	0.0125–0.05 mg/kg every 6–12 months (maximum dose 4 mg)
Alendronate (2nd generation)	Oral	1–2 mg/kg/week < 40 kg: 5 mg/day or 35 mg/week > 40 kg: 10 mg/day or 70 mg/week Maximum dose: 70 mg/week
Risendronate (3rd generation)	Oral	15 mg/week (< 40 kg); 30 mg/week (> 40 kg) Maximum dose: 30 mg/week

The optimal treatment duration is not clearly defined and is currently based on expert recommendations [116]. We propose discontinuing or progressively decreasing BPs dosing for those patients who have not presented fractures during the preceding year and who have attained a Z-score higher than -2.

### Follow-up

The aim of follow-up in patients with osteoporosis risk factors is to identify those candidates who need to start or maintain specific treatments. For such patients and those with an established osteoporosis diagnosis, follow-up should be continued as long as risk factors persist or while treatment is maintained with calcium and/or vitamin D_3_, BPs or other medications for osteoporosis [49, 51, 74].

Clinical, radiological and analytical parameters should be monitored. Assessing the number of fragility fractures and pain episodes is important. In terms of densitometry, variations in Z-scores are relevant. The optimal frequency for DXA performance is insufficiently defined [74]. Our recommendation is to repeat DXA after one year, and then every 1–2 year thereafter according to the patient’s trajectory, with a minimum interval between checks of 6–12 months.

It is also crucial to perform a radiological assessment of VF, since they are frequently asymptomatic and can appear even in patients with Z-scores higher than -2 [23]. Moreover, their evolution can lead to changes in treatment [116]. There are no studies that have definitively determined how often VF should be monitored, although some authors propose lateral spine x-rays on an annual or biannual basis [23]. We propose their frequency be individualized according to the patient’s risk factors, with a minimum period of 6 months and a maximum period of 2 years.

In addition, no studies or guidelines have established the optimal periodicity for assessing phosphocalcic metabolism. Our recommendation is to make an analytical determination on an annual basis.

In regard to patients receiving calcium and vitamin D supplementation, since the optimal intake for children and adolescents suffering from chronic diseases is unknown [41], doses should be modified according to calciuria and plasmatic levels of 25-hydroxyvitamin D_3_ and iPTH. The optimal frequency for monitoring these parameters is unknown [49], although some authors advocate that determinations should be made every 3–12 months [49, 51]. Our working group recommends that levels of 25-hydroxyvitamin D_3_ should be determined every 6–12 months, or after 3–6 months after a dose change. Furthermore, an annual determination of calciuria is recommended. A renal ultrasound should be conducted to rule out nephrocalcinosis in the event of calciuria increase, or when urine collection is not possible.

Regarding children being treated with BPs, there are no studies that have determined an optimal frequency for analytical checks. Our group recommends monitorization prior to each infusion for patients receiving intravenous BPs, and every 6 months for patients taking BPs orally.

#### Corticosteroid-induced osteoporosis

GCs are widely used with a proven effectiveness in numerous pediatric diseases. However, they carry multiple side effects, and are associated with decreased BMD and bone fragility fractures [118].

Patients treated with systemic GCs lose bone mass more markedly during the first 3–6 months of treatment, mainly trabecular bone [118]. This loss depends on the dose and treatment duration [119, 120]. Although lower doses are less harmful than higher doses, there appears to be no unequivocally safe dose since fracture risk have been reported to persist with prednisone (or equivalent) doses of 2. 5 a 7. 5 mg/ day [118].

Thus, as in other patients with osteoporosis risk factors, monitoring BMD and VF occurrence is advisable. In the absence of clear data on the optimal time for a DXA in this group, we recommend performing a DXA during the first 6 months of treatment, and repeating it every 9-12 months if treatment continues.

Regarding VF screening, some studies reported an incidence rate of around 10% during the first year, with nearly 50% of such cases being asymptomatic [70, 71]. For this reason, it is advisable to assess these patients using imaging technique at the beginning of treatment, and thereafter annually while GCs are maintained.

The Spanish Rheumatology Society Consensus [121] holds that the prevention of GIOP must begin as early as possible for all patients receiving doses higher than 5 mg/day of prednisone (or equivalent) for more than three months. Preventive actions include prescribing the lowest possible dose of GCs to control the underlying disease, as well as encouraging physical exercise, avoiding toxic products, such as tobacco and alcohol, and ensuring a balanced diet with the required intake of calcium and vitamin D [121]. In fact, a recent systemic review concluded that calcium and vitamin D supplementation should be started with the same dose recommended for healthy children in all children on GCs, particularly when treatment is expected to last more than 3 months, as a preventive action against GIOP development [95]. In addition, our group recommends maintaining this supplementation for three months after discontinuation of GCs treatment since its effect on bone continues even after treatment has been halted. Nevertheless, no studies have determined an optimal period of supplementation. This same review recommends the use of BPs for preventive purposes [95], despite the lack of any comprehensive data. Our own working group does not consider its systematic use in the absence of fragility fractures. Nevertheless, its effectiveness is proven when GIOP has been established; i.e., when pathological fractures are clearly evident [87, 95, 96].

Most studies suggest that an inhaled GCs dose lower than the equivalent of 800 mcg/day of budesonide has only a minimum effect on fracture risk, while higher doses are associated with an accelerated decrease in BMD and a higher risk of fractures. In these patients, although non-pharmacological preventive actions are justified [97, 98, 122], it is not advisable to routinely carry out such procedures as lateral spine x-rays or DXAs, unless these patients have other risk factors [97–99]. Furthermore, the role of calcium and vitamin D supplementation in patients prescribed inhaled GCs has not yet been established, although some groups recommend supplementation for higher risk populations [93].

## Conclusion

In summary, we present herein guidelines for the prevention, diagnosis and treatment of secondary childhood osteoporosis based on the available evidence and expert clinical experience. We believe it can serve as a useful tool that will contribute to the standardization of clinical practice for this pathology. Prophylactic measures, early diagnosis and a proper therapeutic approach are essential to improving bone health, not only in children and adolescents, but also in the adults they will become in the future.

## Data Availability

Not applicable.

## Abbreviations

*BMC*: Body mass content
*BMD*: Body mass density
BPs: Bisphosphonates
CEBM: Centre for Evidence-based Medicine
CTx: Carboxy-terminal telopeptides
DXA: Dual-energy x-ray absorptiometry
GCs: Glucocorticoids
GIOP: Glucocorticoid-induced osteoporosis
iPTH: Intact paratohormone
P1NP: Amino-terminal propeptides from type 1 procollagen
SERPE: Pediatric Rheumathology Spanish Society
UVB: Ultraviolet B
VF: Vertebral fractures
